# Supplementary material for: Acceptance of AI-based tools in consumer financial decision-making: An application of the extended technology acceptance model
Source: PLoS One. 2026 Mar 19;21(3):e0344901. doi: 10.1371/journal.pone.0344901 (PMC13001959; doi:10.1371/journal.pone.0344901)
Supplement: Attachment — Submitted filename: Rebuttal letter.docx [file pone.0344901.s001.docx]

**Rebuttal letter**

The authors of the article would like to thank the reviewers for their insightful analysis and comments, which allowed us to significantly improve the quality of our article. We believe that after the changes we have made, reviewers will consider our article to be of appropriate scientific quality. We have carefully considered all the comments that were presented in the reviews and have addressed all of them.

**Reviewer 1**

1. **Novelty and originality of the research must be added to the abstract**

We changed the abstract and added the required text

2. **The introduction is long**

We restructured and significantly changed the introduction as well as cut it in some parts. We did not want to reduce it fundamentally as in our opinion the scientific issue we address is quite complex and requires a proper insight.

3. **The literature review must engage in the constructs of your analytical framing in a meaningful way.**

We developed the theoretical background and added a critique of the literature, focusing in particular on the AI-based tools in consumers’ financial management and not on AI in general

4. **The Discussion lacks a critical synthesis and comparison**

We significantly restructured and rewrote the discussion with the review of prior research

5. The Conclusion does not adequately discuss the theoretical and managerial implications of the study

We significantly changed the conclusions

**Reviewer 2**

1. **Limited Sample Representativeness**

We included additional comments and information addressing the issue of the sample. The classic TAM model does not directly relate to various socio-demographic features. We are conscious of the bias of the research sample, and we introduced such a comment. Our full future research will be based on a representative sample of society.

2. **Superficial Literature Review**

We expanded the literature review and focused more on the AI-based tools in financial decision-making by consumers

3. **Measurement Instrument Concerns**

We added the information about the development of the GAAIS and the loadings of the model.

4. **Methodological Gaps**

We added the explanation of the prolonged data collection procedure. The time frame of the collection process was dependent on the semester’s layout at the universities and the necessity to get a sample of a few hundred respondents. As the data was gathered as a convenience sample from the students participating in the courses provided in the three universities, we are not possible to present the response rates and non-response bias. The purpose of the sample was to test and validate the survey.

5. **Underdeveloped Discussion**

We restructured and fundamentally expanded discussion and added examples of other studies

6. **Overgeneralization of Findings**

We strongly expanded the text related to the sample bias that may impact the findings and added some information about context of Poland.

7. **Clarity and Writing Issues**

We corrected the manuscript with the support of the native speaker and added the descriptions of the figures.

**8. Limited Exploration of Socio-Demographic Factors**

Due to the convenience sampling, we purposely omitted the analysis of the socio-demographic influence in the pilot study. It will be included in the research based on the representative sample.
