# Supplementary material for: Acceptance of AI-based tools in consumer financial decision-making: An application of the extended technology acceptance model
Source: PLoS One. 2026 Mar 19;21(3):e0344901. doi: 10.1371/journal.pone.0344901 (PMC13001959; doi:10.1371/journal.pone.0344901)
Supplement: Attachment — Submitted filename: 21_08_PLOS Rebuttal letter 2.docx [file pone.0344901.s002.docx]

**Rebuttal Letter 2**

Once again, we would like to thank the two anonymous reviewers for their thorough review of our article, pointing out shortcomings and offering suggestions and recommendations to improve its quality. We have taken all your comments into consideration and have tried to revise the article and make changes as closely as possible to your suggestions. All the modifications in the manuscript are indicated in yellow color. We hope that this time we have met the requirements. Thank you again for your scientific support and patience.

Below, we present our response to each of the comments made by the two reviewers.

**Response to REVIEWER 1**

**Introduction**

***Much sharper problematization is required so that the introduction draws the reader into the paper. The introduction needs to do a better job of setting the stage for the articulation of the theoretical contributions of the study. The descriptions of the research philosophy and design adopted in the study need to be sufficiently developed.***

We have significantly restructured the introduction, following the recommendation to use 5 paragraphs. We distinguished subsections concerning what we know, what we don’t know, and why the topic is important, as well as what we learn from the study. We make the introduction more precise by reducing a bit of the general context.

**The discussion**

***The Discussion lacks a critical synthesis and comparison of the primary data with the literature.***

We have restructured the discussion to provide a clearer synthesis and interpretation of the key findings. Now, we are comparing our primary data more explicitly with previous literature, focusing on areas of convergence and divergence.

Additionally, we have expanded the scope of referenced literature to include more recent and thematically relevant studies. This allowed us to contextualize the rejection of hypothesis H4 more effectively and discuss its implications within the broader TAM research field and the specific domain of AI in personal finance.

**The conclusion**

***The Conclusion does not adequately discuss the theoretical and managerial implications of the study. Identify and discuss how a gap in the literature has been addressed and demonstrate the importance of your ideas. Introduce possible new or expanded ways of thinking about the research problem. Also, state the ideas for future research in the conclusion.***

We have restructured and expanded the conclusions section and clearly distinguished three subsections:
(1) Theoretical Implications,
(2) Managerial and Policy Implications, and
(3) Directions for Future Research.

**Response to REVIEWER 2**

**Sample representativeness and generalizability**

***The manuscript does not report response rates or non-response bias, which are critical for assessing the representativeness of even a convenience sample. Expand the Limitations section (Page 78) to discuss how the sample’s homogeneity (young, digitally literate students) may skew perceptions of AI tool acceptance. Provide an estimate of response rates or explain why this was not feasible. Clarify how the planned representative sample study will address these limitations.***

We have added a paragraph related to the sample homogeneity bias issue in the materials and methods section with the references, as well as expanded the description in the limitations section. We have also included consideration of how the representative sample may relate to the results obtained from the current sample.

Due to the specific goal of our investigation, which was validation of the research questionnaire, verification of the proper understanding of the questions, and analysis of the relations between the main constructs, we did not focus on the impact of socio-demographic features. Also, the simplified sampling technique, which we used, did not give us a basis for deep socio-demographic considerations. Therefore, we decided to keep the description of the socio-demographic aspects rather limited to maintain the proper structure of the text (focusing on the aspects of perception, attitudes, intentions, and actual use of AI)

Because we collected the data from the respondents through the research questionnaire, which was accessed by the QR code placed on the websites of the three university faculties that took part in the research, we cannot express the precise response rate. We could use the number of responses divided by the total number of students per faculty, but that does not necessarily show the proper rate (not all the students are browsing the faculty website, and there could be other respondents, such as post-graduate students, professionals, and graduates who can do so). Sending the research questionnaire by email would give us the possibility to calculate the response rate, but according to our experience, survey distribution by email is even less effective for collecting data (students usually do not respond to such emails).

**Depth of literature review**

***The literature review (Pages 15–18) is comprehensive but could better engage with recent studies on AI in consumer finance beyond robo-advisors. For example, the discussion of general conversational AI models (e.g., ChatGPT, Claude) is brief and lacks integration with specific findings (e.g., Lopez-Lira & Tang, 2023).***

***The review does not sufficiently critique the limitations of prior studies, such as their focus on institutional perspectives or specific AI applications (e.g., chatbots, voicebots).***

We significantly expanded the literature review section, inserting new references.

**Methodological details**

***The prolonged data collection period (February to October 2024) is explained as due to academic semesters and summer breaks (Page 20), but the manuscript does not discuss potential temporal effects (e.g., changes in AI adoption trends during this period).***

***The adaptation of the General Attitudes Towards Artificial Intelligence Scale (GAAIS) is mentioned (Page 14), but details on how it was tailored to the financial decision-making context are lacking.***

***The decision to retain items with factor loadings below 0.7 (Page 68) is justified, but the manuscript could clarify why these items were theoretically important to retain.***

We have added a detailed explanation of the process of the scale adaptation in the section Materials and Methods. We have clearly expanded the theoretical justification for retaining items with lower factor loadings according to the Reviewer’s recommendation.

In the revised manuscript, we have expanded our explanation to clarify why the four retained items—PU7, PU8, PU12, and ATT7—were preserved despite factor loadings slightly below 0.7. While their removal did not improve internal consistency, composite reliability (CR), average variance extracted (AVE), or reduce variance inflation factors (VIF), our decision was grounded not only in statistical evaluation but also in the unique theoretical value of these items.

Each of them captures important aspects of consumer perceptions regarding AI-based tools in financial decision-making that are not redundantly represented by other items in the scale. Specifically, they address perceived benefits such as risk reduction, improved management of savings, rapid access to financial data, and AI’s potential superiority in certain financial tasks. These dimensions are integral to the constructs of perceived usefulness and attitude and align directly with the conceptual framework and research gap addressed in our study. Removing them would have compromised the content validity and the conceptual breadth of the model.

**Discussion and theoretical implications**

***The Discussion section (Pages 74–77) effectively compares findings with prior studies (e.g., Belanche et al., 2019; Singh & Kumar, 2025) but could further explore the theoretical implications of the rejected H4 (PU → BI). This finding challenges the traditional TAM and warrants deeper analysis of why perceived usefulness does not directly influence behavioral intention in the AI context.***

***The discussion of mediation effects (H7, H8) is clear but could be expanded to compare with other TAM-based studies in finance (e.g., mobile banking, QR payments).***

We have restructured the section to provide a clearer synthesis and interpretation of the key findings. We are comparing more explicitly our primary data with previous literature - we have added over a dozen references from the years 2023–2025.

**Clarity and writing quality**

***The manuscript is generally clear but contains minor grammatical errors and awkward phrasing (e.g., “in contrary” on Page 71 should be “in contrast”).***

The grammatical errors were checked by the native speaker. We hope all the errors were removed.

***Figures 1–3 are referenced but not embedded in the provided document, making it difficult to evaluate their content and clarity.***

Unfortunately, we have no possibility to insert the figures into the main text of the manuscript. According to the PLoS One publishing rules, the figures must be added as separate files – what we do when uploading the manuscript and its revised versions.

***The Conclusions section (Pages 77–79) is concise but could better articulate the study’s contributions to both theory (e.g., extending TAM) and practice (e.g., specific design recommendations for AI tools).***

We have significantly expanded the conclusions sections and clearly distinguished its three parts:
(1) Theoretical Implications,
(2) Managerial and Policy Implications, and
(3) Directions for Future Research.

**Specific comments**

***Abstract (Page 9): The abstract is concise but could better emphasize the study’s novelty. Consider adding a sentence on how the findings challenge traditional TAM assumptions (e.g., rejection of H4).***

We have modified the abstract to better emphasize the study’s novelty and the findings.

***Introduction (Pages 10–14): The introduction is well-structured but could be streamlined by reducing repetitive references to AI’s general capabilities. Clarify why Poland is a unique context for this study (e.g., digital adoption trends, financial literacy levels).***

We have reduced the Introduction by cutting the part of the general context of the AI. Moreover, we have added a short comment about Poland, explaining the country’s potential for the implementation and development of innovations in finance.

***Literature Review (Pages 15–18): The hypotheses are well-supported, but the discussion of conversational AI tools (e.g., ChatGPT) could cite more recent studies to strengthen relevance.***

We have clearly developed the literature review section and added new references to strengthen relevance.

***Methods (Pages 19–25): The demographic breakdown (Page 20) is detailed, but a table summarizing respondent characteristics would improve readability.***

The table has been added.

***Results (Pages 67–73): The rejection of H4 (PU → BI) is a key finding and should be highlighted more prominently in the Discussion as a contribution to TAM literature.***

We have expanded the interpretation of the rejected H4 hypothesis, highlighting its theoretical significance within the TAM framework. The revised section discusses how the full mediation of perceived usefulness through attitude challenges conventional TAM assumptions and opens new theoretical perspectives for AI-related contexts. We restructured the Conclusions section, inserting more details of our contributions to theory, managerial policy implications, and directions for future research.

***Discussion (Pages 74–77): The comparison to prior studies is adequate but could include more examples of AI tools (e.g., Finchat, TurboTax) to illustrate practical implications.***

We have restructured the Discussion section to provide a clearer synthesis and interpretation of the key findings. We now more explicitly compare our primary data with previous literature. We have also added over a dozen references from the years 2023–2025. Additionally, we slightly repositioned the examples of consumer-facing AI applications (e.g., Finchat, TurboTax Assistant, Cleo, Emma), now using them as illustrative support for our empirical findings rather than as a standalone contextual note. This helps to strengthen the practical implications of the observed mediation effect by showing real-world relevance.

***References (Pages 80–84): The reference list is comprehensive, but some DOIs are formatted inconsistently (e.g., “doi-1org-100bc2fxf0579.han3.uci.umk.pl”). Ensure all references follow PLOS ONE guidelines.***

We have revised with care the references again and corrected when needed. The one “doi-1org-100bc2fxf0579.han3.uci.umk.pl” is exactly as it is provided by the Journal that published the article.

Sincerely,

Tomasz Szopiński, Michał Buszko, Małgorzata Porada-Rochoń
