# Supplementary material for: Acceptance of AI-based tools in consumer financial decision-making: An application of the extended technology acceptance model
Source: PLoS One. 2026 Mar 19;21(3):e0344901. doi: 10.1371/journal.pone.0344901 (PMC13001959; doi:10.1371/journal.pone.0344901)
Supplement: Attachment — Submitted filename: 25_11_PLOS Rebuttal letter 3.docx [file pone.0344901.s003.docx]

**Rebuttal Letter 3**

Once again, we would like to thank the reviewers for their thorough review of our article, which highlighted shortcomings and provided valuable recommendations for its improvement.

We have taken all the comments into consideration and have attempted to revise the article, making changes according to all the indications. All improvements to the manuscript are highlighted in yellow. Moreover, we reviewed the whole text once again and introduced minor linguistic and stylistic adjustments. We hope that these improvements have met the postulated requirements.

Thank you again for your scientific support!

Below, we present our response to each of the comments

**Response to REVIEWER 1**

**The discussion lacks a critical synthesis and comparison of the primary data with the literature**

We rewrote the discussion section almost from scratch, interpreting and describing the significance of our findings in relation to the existing literature and to the latest knowledge about our research problem. We have also explained new insights that emerged from our research (primarily the attitudes mediation channel).

**The discussion should include a critical synthesis and comparison of the data with the literature**

We supported our discussion section, particularly in verifying hypotheses, with literature sources, and made a critical synthesis. Each hypothesis discussion is divided into clear subsections.

**The conclusion does not adequately discuss the theoretical and managerial implications of the study**

We rewrote the conclusion section almost from scratch, summarizing the paper's key points and emphasizing the significance of our research. We also addressed the gap in literature coverage and underlined the importance of our ideas. Moreover, we have created clear subsections of the Conclusion part, including Theoretical Implications, Managerial Implications, Limitations, and Ideas for Future Research

**Response to REVIEWER 2**

Thank you very much for the positive feedback and acceptance of the paper!

*Tomasz Szopiński, Michał Buszko, Małgorzata Porada-Rochoń*
